# Salty sisters: The women of halophiles

**DOI:** 10.3389/fmicb.2014.00192

**Published:** 2014-06-04

**Authors:** Bonnie K. Baxter, Nina Gunde-Cimerman, Aharon Oren

**Affiliations:** ^1^Great Salt Lake Institute, Westminster CollegeSalt Lake City, UT, USA; ^2^Molecular Genetics and Microbiology, University of LjubljanaLjubljana, Slovenia; ^3^Centre of Excellence for Integrated Approaches in Chemistry and Biology of ProteinsLjubljana, Slovenia; ^4^Department of Plant and Environmental Sciences, The Institute of Life Sciences, The Edmond J. Safra Campus, The Hebrew University of JerusalemGivat Ram, Israel

**Keywords:** halophiles, women in science, diversity, history of science, Nobel Prize

## Abstract

A history of halophile research reveals the commitment of scientists to uncovering the secrets of the limits of life, in particular life in high salt concentration and under extreme osmotic pressure. During the last 40 years, halophile scientists have indeed made important contributions to extremophile research, and prior international halophiles congresses have documented both the historical and the current work. During this period of salty discoveries, female scientists, in general, have grown in number worldwide. But those who worked in the field when there were small numbers of women sometimes saw their important contributions overshadowed by their male counterparts. Recent studies suggest that modern female scientists experience gender bias in matters such as conference invitations and even representation among full professors. In the field of halophilic microbiology, what is the impact of gender bias? How has the participation of women changed over time? What do women uniquely contribute to this field? What are factors that impact current female scientists to a greater degree? This essay emphasizes the “*her* story” (not “*his*tory”) of halophile discovery.

## INTRODUCTION

Women’s participation in science was historically limited as the knowledge-creators were traditionally men in most civilizations. Women’s societal roles made careers more challenging in general, and science was seen as a demanding career. But many of us working in science today are surprised to learn the level of gender bias that persists even now, and perhaps we have reached a point to challenge the alarming statistics.

Though representation varies across fields, women are awarded about half of science doctorates in the US, but only 21% of full science professors are women (National Science Foundation, National Center for Science and Engineering Statistics, 2013). This issue affects not only job opportunities, but also one’s purse as female scientists earn only 82% of that of males in the US, and this figure is even lower in Europe (European Gender Summit, 2012). Other studies, such as one among chemistry graduate students in the UK, have looked at the social factors that disproportionately affect women such as family choices (Royal Society of Chemistry, 2008). Furthermore, disparities in promotion, grant-funding and tenure persist (Shen, 2013).

In Europe, a commission of scientists is working on this issue. A recent congress identified priority areas that may impact the level of participation of women in these fields (European Gender Summit, 2012). These include active recruitment and retention, assessment and value systems. Much focus of this commission is on changing institutional practices and processes to result in more balance in human resources.

Whatever the factors that impact women’s participation, it is clear that bias also exists, and there is evidence that this bias, at least in conference participation, may be at the front end: the invitation. Skewed ratios of men to women lecturers at conferences have been shown in several recent studies. Isbell et al. (2012) published an analysis of speakers in the female-heavy field of primatology, Schroeder et al. (2013) quantified the number of female invitees and speakers at evolutionary biology conferences, and Eisen (2012) reported on the number of talks given by women at quantitative biology meetings. All three studies used powerful statistics to compare the speakers to baseline populations of participants in their field of study. Which factors caused the biases observed? Schroeder et al. (2013) concluded that that fewer women were among the invitees and/or women turned down invitations at a higher rate. A preference for invited male scientist authors was also discovered in a review of “News & Views” articles in *Nature* and “Perspectives” in *Science* for 2010 through 2011 (Conley and Stadmark, 2012), prompting the journals to analyze the underrepresentation in their practices of author invitation.

If there is bias at the level of invitation, then we should look closely at those who do the inviting. The organizing committee of any conference typically is charged with the structure of the meeting and the invited speaker list. An analysis of 460 symposia supported by the American Society for Microbiology demonstrated that at least one female scientist on the organizing committee resulted in a greater number of invited speakers who were women (Casadevall and Handelsman, 2014).

Thus, the issue of the participation of women in many aspects of scientific life has garnered some attention in a number of forums. The authors of this study are halophile scientists and participants in the triennial international halophile congresses. We became curious about the gender balance within this field and set out on a study to analyze the representation and participation of women in high-salt microbiology. The results were presented at the Halophiles 2013 conference in Storrs, CT, USA, and they are now reported here. Most importantly, we also highlight some of the most prominent female scientists in the field of halophilic microorganisms.

## MATERIALS AND METHODS

We obtained the programs from each conference on halophiles from 1978 to the recent meeting in 2013 (**Table 1**; Oren, 2011). In looking for historically important and noteworthy women halophile scientists, we looked for early participants, multiple conference participants, contributions to the field, and female lectures who were invited to speak. We also collected information about important early discoveries in the field that were attributed to women.

**Table 1:** A compilation of all international Halophile conferences, locations and an indication of the participation of women.

Year	Conference title	Location	# Women	# Total
1978	European Molecular Biology Organization (EMBO) Workshop on Halophilism	Rehovot, Israel	^*^8	^*^56
1985	^**^The Molecular Basis of Haloadaptation in Microorganisms	Obermarchtal, Germany	^*^2	^*^21
1986	Aspects of Halophilism	Jerusalem, Israel	^*^8	^*^39
1989 (March)	Modern Aspects of Halophilism	Neve Ilan and Rehovot, Israel	^*^22	^*^57
1989 (September)	^**^General and Applied Aspects of Halophilic Microorganisms	Alicante, Spain	^*^23	^*^65
1992	American Society for Microbiology (ASM): Halophilic Bacteria	Williamsburg, VA, USA	^*^6	^*^34
1997	Microbiology and Biogeochemistry of Hypersaline Environments	Jerusalem, Israel	^*^13	^*^69
2001	International Conference on Halophilic Microorganisms	Seville, Spain	8	61
2004	^**^Halophiles 2004	Ljubljana, Slovenia	17	57
2007	^**^Halophiles 2007: International Congress Exploring Life at High Salinity	Colchester, UK	16	49
2010	Halophiles 2010: International Conference on Halophilic Microorganisms	Beijing, China	15	48
2013	^**^Halophiles 2013	Storrs, CT, USA	27	59

* The value is numbers of female participants and total participants. From 2001 forward, all values are numbers of female oral presenters and total lectures.

** The conferences had at least one female convener on the organizing committee.

For examination of current researchers, female lecturers, who had a track record in publications in halophile science and were invited to speak at conferences from 2001 forward, were asked to self-report their main achievements in the field of halophilic microorganisms and to select references that they consider most significant. In some cases, we edited the text for length or consistency.

To get an idea of participation of women at these conferences, in the programs of meetings before 2001, we counted the number of female participants as the programs did not distinguish between oral versus poster presentations and the numbers of total participants were low. In 2001, the programs were organized to show oral talks, and for these conferences through the most recent one, we counted the numbers of women who gave oral lectures. Comparing this number of women to the total number of presentations, we computed a percentage of females for each conference. If the gender was not discernable (e.g., initials were used), then we did not count the person in the total.

## RESULTS

### HISTORICAL CONTRIBUTORS

When, in the days of Abraham the Patriarch during the destruction of the cities of Sodom and Gomorrah, Lot’s wife turned into a pillar of salt on the shore of the Dead Sea, as described in Genesis 19: 26, she sure did not have much opportunity to marvel at the interesting halophilic microorganisms inhabiting the lake. The interesting properties of organisms such as *Haloarcula marismortui*, recovered from the Dead Sea by a woman scientist (Ginzburg et al., 1970; Oren et al., 1990), were discovered only three millennia later.

Here is a selection of women-scientists who devoted efforts to the elucidation of the properties of halophilic life. It should be noted that these women were pioneers and worked in an age of extreme underrepresentation of women in science. Our selection is based on the historical importance of their contributions to the field in the period up to the end of the 20th century.

#### Clara Hamburger

Flagellated unicellular algae colored orange-red by their high content of β-carotene were first documented in saltern brines by Dunal in 1838, who named the organism *Haematococcus salinus*. Two detailed descriptions of the organism were published in 1905, one by Clara Hamburger from Heidelberg and one by E. C. Teodoresco in Bucharest. Clara Hamburger’s study is by far the most detailed. She provided a summary of the debate, still unresolved at the time, about the cause of the red-purple or red-orange coloration of saltern ponds approaching salt saturation. Unfortunately for her, the formal description of the genus *Dunaliella* and the species *Dunaliella salina* by Teodoresco (1905) preceded the publication of Hamburger’s (1905) study by a few months, as she describes herself in her article. Hamburger’s observations about the location of the β-carotene in small globules within the single chloroplast were surprisingly exact, and she criticized the incorrect statements of Teodoresco on this subject: “It occurs in the form of small droplets, and is, as seems sure to me, only deposited in the outer alveolar layer of the plasma, while the chromatophore [= chloroplast] is the bearer of the green pigment. The remark by Teodoresco ‘the blood pigment that impregnates not only the chromatophore, but also the whole body of adult individuals’ does not correspond with my observations.” (translation: Aharon Oren).

#### Helena Petter and Trijntje Hof

Among the group of microbiologists associated with the “Delft School” in the 1920s–1930s were two women who wrote their Ph.D. thesis on halophilic microorganisms and contributed much to our understanding of the halophiles: Helena Petter and Trijntje Hof. In her thesis (University of Utrecht) “Over roode en andere bacterieën van gezouten visch” (On red and other bacteria of salted fish; Petter, 1931, 1932), Helena Petter studied a variety of different halophilic prokaryotes, most of them red pigmented members of the *Halobacteriaceae*, isolated from salted fish and from “Trapani” salt used to preserve fish, which she obtained from a cannery in Bergen, Norway. Her isolates included rod-shaped bacteria as well as coccoid and sarcina-shaped form. Her studies include a description of “Bacterium halobium” (currently known as *Halobacterium salinarum*). The name bacterioruberin for the carotenoid pink pigment of the *Halobacteriaceae* was first introduced by Petter. She made drawings of the gas vacuoles within the cells, and she was the first to explain the possible ecological advantages of these gas vacuoles and of the buoyancy these gas vesicles bestow upon the cells: in hypersaline lakes in which only little oxygen can dissolve, there may be a selective advantage for oxygen-dependent microorganisms to float toward the surface of the brine when oxygen becomes limiting in the deeper layers.

Trijntje Hof’s thesis (University of Leiden) on “Investigations concerning bacterial life in strong brines” (Hof, 1935) included a description of a novel type of motile halophilic rod that caused a purple discoloration of salted beans. She named this organism *Pseudomonas beijerinckii* in honor of Martinus Beijerinck, the founding father of the Delft school of microbiology. *P. beijerinckii* was renamed as *Chromohalobacter beijerinckii* in 2006.

#### Arlette Danon

Arlette Danon of the Weizmann Institute of Science, Rehovot, Israel, started work on the function of the purple pigment bacteriorhodopsin (BR) of *Halobacterium* very soon after the chemical structure of the pigment was discovered in the early 1970s. The discovery that light energy absorbed by BR in the purple membrane of *Halobacterium* is conserved by photophosphorylation with the formation of ATP (Danon and Stoeckenius, 1974; Danon and Caplan, 1976) is the key to the understanding of the function of the pigment. She also discovered that in the light *Halobacterium halobium* (*salinarum*) cells can fix a certain amount of CO_2_, mediated by BR (Danon and Caplan, 1977). The mechanism of this light-dependent CO_2_ fixation, later studied by another woman halophile scientist – Barbara Javor – is still not completely clear.

Danon passed away at an early age, ending a short but very promising career in halophile science.

#### Margot Kogut

“In the early 1950s … I can still see in my mind’s eye the lady who worked at the bench adjacent to mine, Margot Kogut, standing there in white lab coat and scarf huddled over a cup of hot tea.” Thus described John Ormerod (2003) his memories of Margot Kogut of University of London Kings College. Margot studied the salt relationships of moderately halophilic Bacteria, using *Salinivibrio* costicola as the model organism. Her studies of in vitro protein synthesis by its ribosomes, in collaboration with Donn Kushner’s group in Canada (Wydro et al., 1977) led to a proper assessment of the true intracellular environment of a moderate halophile that can adapt to a broad range of salt concentrations. Her studies of the lipids in the cell membrane and the changes in lipid composition as a function of the salinity of the medium (Russell and Kogut, 1985; Russell et al., 1986) were used to confirm and to illustrate her ideas about the difference between “adaptation” to extreme environments and “adaptability” toward changes in the parameters determining the extreme habitat (Kogut, 1980a, b).

Together with Bill Grant (Leicester), Kogut organized the 1985 symposium on ‘The molecular basis of haloadaptation in microorganisms’ in Obermarchtal, Germany (Grant and Kogut, 1986). This meeting can be considered as the first congress on all aspects of halophilic microorganisms, the Rehovot 1978 symposium being almost entirely dedicated to the biochemistry of BR.

#### Barbara Javor

Barbara Javor received her Bachelor of Arts degree from the University of California Santa Barbara, and her Ph.D. in Biology at the University of Oregon. She spent a post-doctoral period to study the salterns of Eilat at the Red Sea coast of Israel, and has worked on halophilic microorganisms at Scripps Institution of Oceanography, University of California. Later she joined different biotechnological companies in the San Diego, CA, area.

Among halophile scientists she is renowned for her textbook “Hypersaline environments.” Microbiology and biogeochemistry (Javor, 1989). This famous and important monograph explores all aspects of natural hypersaline lakes, salterns, saline soils and other salt-stressed environments and the microorganisms inhabiting them. She has explored the prokaryote diversity of salterns in different locations worldwide (Javor, 1983, 1984), and isolated interesting “box-shaped” halophilic Archaea, which turned out to belong to the genus *Haloarcula* (Javor et al., 1982). She also continued the study of the light-dependent CO_2_ fixation (Javor, 1988), a topic initiated by Danon a decade earlier (see above).

Some of her experiences as a consultant for salt production companies and on the links between the biology of the saltern ponds and the quantity and quality of salt produced are summarized in her paper on industrial microbiology of solar salt production (Javor, 2002).

#### Margaret Ginzburg

Margaret Ginzburg (the Hebrew University of Jerusalem) has published a large number of papers, many of which were prepared jointly with her husband Ben-Zion Ginzburg, both on halophilic Archaea and on *Dunaliella*.

After the original isolate of *Halobacterium marismortui* retrieved from the Dead Sea by Benjamin Elazari-Volcani had been lost, Ginzburg et al. (1970) isolated and characterized a very similar strain from the lake, and described it as *Haloarcula marismortui* (Oren et al., 1990). This isolate served as the object for many studies on the ion metabolism, the permeability properties of the membranes, and the bioenergetics of halophilic Archaea, many of which also included comparisons with *Halobacterium salinarum*, found to behave differently in certain aspects (Ginzburg, 1969, 1978; Ginzburg et al., 1970). Ion metabolism and the metabolism of the osmotic solute glycerol were studied in different *Dunaliella* isolates, including strains obtained from the Dead Sea (Ginzburg and Ginzburg, 1985), and her studies about *Dunaliella* led her to write a long review about the adaptation of *Dunaliella* to life at high salt concentrations (Ginzburg, 1987). Caplan and Ginzburg (1978) edited the proceedings volume of the above-mentioned symposium on “Energetics and structure of halophilic microorganisms” held in Rehovot and dedicated mainly to the properties of BR.

#### Rita Colwell

Rita Colwell, the first female Director of the U.S. National Science Foundation, is well known for her efforts to increase the participation of underrepresented groups in science. During her career as a microbiologist, she studied *Vibrio cholerae* and other slightly halophilic *Vibrio* species, focusing on the role of the environment and climate change in disease dynamics. But some of Colwell’s studies were devoted to prokaryotes living at higher salt concentrations. Her 1979 taxonomic study on red halophilic Archaea, performed jointly with Carol Litchfield (see below) and other coworkers, provided a polyphasic comparative study of the then-known, limited diversity of the *Halobacteriaceae* (Colwell et al., 1979). Rita also is a member of the International Committee on Systematics of Prokaryotes (ICSP) Subcommittee on the Taxonomy of *Halobacteriaceae*. More recently her group has described a new species of *Halobacillus*, *Halobacillus thailandensis*, from fish sauce (Chaiyanan et al., 1999).

#### Carol Litchfield

Carol Litchfield had a great fascination for salt lakes and the organisms inhabiting them. “Red – the magic color for solar salt production” (Litchfield, 1991) was the starting point for many of her studies on halophiles in solar salterns world-wide, in Great Salt Lake, Utah, and in other hypersaline aquatic environments. With her student, Russell Vreeland, she discovered and described *Halomonas elongata* from a saltern pond on Bonaire (Vreeland et al., 1980), an organism that since has become the model organism for the study of moderate halophiles, and that, thanks to the production of ectoine, has also a great biotechnological interest. Litchfield extended her interest in halophiles on Earth to the possibility that such organisms may inhabit or may have inhabited other planets in the past (Litchfield, 1998). She applied techniques such as polar lipid analysis and pigment analysis to the comparative characterization of the biota of saltern ponds and salt lakes (Litchfield et al., 2000), and she also pioneered combining cultivation of the halophiles along with molecular techniques (Litchfield and Gillivet, 2002). She was an active member of the ICSP Subcommittee on the Taxonomy of *Halobacteriaceae*. Interestingly, Litchfield also was an amatuer salt industry historian, and she co-edited a book of essays on the history of salt and salt-making (Litchfield et al., 2001). She was an avid collector of old books and documents on salt as well as artifacts connected with salt production. Her extensive collection of is now at the Hagley American Industry Museum in Delaware.

#### Ada Yonath

In 2009, Ada Yonath was the first woman in 45 years to win the Nobel Prize in Chemistry and the first Israeli woman to win a Nobel Prize. A member of the faculty of the Weizmann Institute of Science in Rehovot, Israel, she is a crystallographer best known for her pioneering work on the structure and function of ribosomes. In many of her studies that gained her the Nobel Prize, the Dead Sea archaeon *Haloarcula marismortui* served as the model organism (Makowski et al., 1987; von Böhlen et al., 1991; Schlunzen et al., 1999; Yonath, 2002a, b). In a short essay recently published in *Nature* (Yonath, 2011), Yonath expressed her feelings and ideas about being a female scientist and about the long way to success. She also discussed there the special position of women in science: “*I think science is gender-independent*, ” and “*I’m trying to change the image of scientists, especially of female scientists, so there will not be so many anti-female sentiments.*” We all hope that Yonath’s example will be followed by many other women who will discover the fascinating world of the halophilic microorganism and the opportunities these organisms offer as model systems to answer basic questions in biology.

#### Ada Zamir

Ada Zamir, who joined the faculty of the Weizmann Institute of Science in Rehovot, Israel, in 1964, used *Dunaliella salina* as the model organism for her studies on defense mechanisms of algae against high light intensities and high salinity. She identified a number of membrane proteins induced by *Dunaliella* as a reaction to salt stress: a special carbonic anhydrase and a novel transferrin-like protein (Sadka et al., 1991; Fischer et al., 1996, 1997; Bageshwar et al., 2004). Zamir also studied the molecular mechanisms responsible for the photoinduction of massive β-carotene accumulation by *D. salina* (Lers et al., 1989).

### EXAMPLES OF ACTIVE AND NOTEWORTHY CONTRIBUTORS

To represent the breadth of current female researchers in halophilic microbiology, we relied on the programs from conferences listed in **Table 1**. We recognize the important work in contributed oral presentations and posters by all women since 1978, but for this analysis we focused only on those women who were listed in the programs as “invited speakers.” In addition, these women were filtered for those who had a long-standing commitment to the halophile science demonstrated by years of involvement and publication record. We asked these scientists, who were still working in the field, to self-report their achievements. Those who responded are listed below, in alphabetical order, as examples. Included are their research interests and references for their work. This method resulted in what is certainly not an exhaustive list, but it should serve underscore the participation of women in this area of science.

#### Josefa Antón

One of Antón’s main achievements is the development of fluorescence in situ hybridization (FISH) protocols for analyzing microbial communities inhabiting hypersaline environments. This technique was instrumental in the discovery of the abundance of extremely halophilic bacteria in close-to-saturation environments, and, more specifically, in the discovery of *Salinibacter ruber*, that turned out to be an ecologically relevant extremely halophile. This discovery was followed by a wealth of studies on the diversity and biogeography of this species, as well as genomic studies. Antón is still working on the microdiversity of this and other extremely halophilic Bacteroidetes. Another focus of her research is the study of yet uncultured halophilic viruses by cloning their complete genomes into fosmids. This work intiated the work on environmental haloviruses, which is continuously growing incorporating new approaches and technologies (Representative references: Antón et al., 1999, 2000; Santos et al., 2007).

#### Bonnie K. Baxter

The microbial foundation of Great Salt Lake, an iconic hyper saline environment, lay virtually unexplored until Baxter began working at the lake in 1998. Baxter reached out to halophile scientists and created collaborations to build an understanding of lake microbial communities. Her projects range from microbial diversity (including bacteria, archaea, viruses, algae, and fungi) to the biogeochemistry of stromatolites to ancient biological molecules in halite. What we now know is that this enormous lake has many micro-niches, all of them with their own microbial communities. Her work has shown that this lake is stratified vertically, horizontally, and temporally. Weaving public outreach into her research, Dr. Baxter turned this unique approach to science into “Great Salt Lake Institute,” which facilitates research and education on this unique body of water (Representative references: Baxter et al., 2005; Griffith et al., 2008; Meuser et al., 2013).

#### Kathleen C. Benison

Benison has made contributions to the understanding of extremophile life and fossilization in acid saline lake and groundwater systems, with a focus on ephemeral lake settings in Western Australia, as well as their ancient counterparts in the Permian redbeds and evaporites of the North American midcontinent. She applied the perspective of a geologist and geochemist, linking water chemistry, chemical sediments, and microorganisms and their temporal dynamics in relation to flooding, evaporation, and desiccation stages of these extreme lakes and shallow groundwaters. Benison showed that prokaryotes, algae, suspect fungi, and certain organic compounds can be easily trapped and well-preserved in halite and gypsum, both as solid or fluid inclusions. Together with Melanie Mormile, she has described, for the first time, diverse microbiological communities that thrive in acid brines, with pH as low as 1.5 and salinities up to 32% total dissolved solids. Most of the very diverse organisms are novel. This work has also implications for the understanding of life on early Earth and on other planets, in particular on Mars (Representative references: Benison et al., 2008; Mormile et al., 2009; Conner and Benison, 2013).

#### Angela Corcelli

Corcelli’s studies on halophiles at the University of Bari, Italy, center on two topics: the membrane lipids of extreme halophiles and the properties of BR and the lipids interacting with the BR proton pump in the purple membrane of *Halobacterium*. She discovered a variety of cardiolipins in halophilic Archaea and investigated their function. She also elucidated the structure of the unique sulfonolipids of *Salinibacter ruber* and related members of the *Bacteroidetes*. Her “lipidomics” studies extend from pure culture studies of model halophilic organisms to the characterization of the lipids present in complex communities of halophiles in their natural environment (Representative references: Corcelli et al., 2004; Lopalco et al., 2011; Lobasso et al., 2012).

#### Jocelyne DiRuggiero

Dr. DiRuggiero’s scientific interests are in the adaptations of extremophiles to environmental stresses and in the microbial ecology of extreme environments, in particular environments with extremes in temperature, high salt concentrations, and hyper-arid conditions. Her major contributions to the field of halophiles have been in the elucidation of stress responses of model halophilic archaea to radiation and oxidative stress. Using a combination of functional genomic and genetics she discovered a shift in the archaea away from the eukaryotic model of homologous recombination repair of DNA double-strand breaks. Investigating oxidative stress, and the deleterious effect of ionization radiation, she discovered that non-enzymatic antioxidant processes are essential for the high level of radiation resistance found in *Halobacterium*. More recently, her work in environmental microbiology revealed the ecology of one of the most halophilic environments on Earth. DiRuggiero found that halite pinnacles (NaCl rocks) from the Atacama Desert are inhabited by a photosynthetic-based, archaea-dominated community; she discovered that the diverse prokaryotic assemblages are associated with novel algae related to oceanic picoplankton (Representative references: Kish and DiRuggiero, 2008; Robinson et al., 2011, 2014).

#### Christine Ebel

Ebel’s studies focused on the stability and composition of halophilic enzymes. Her interdisciplinary approach is unique as she combines molecular biology, cellular biochemistry, structural studies, biophysical chemistry, and thermodynamics. Enzymes from halophilic archaea are also halophilic as they have a requirement for high salt. Ebel explores the effects of salts on halophilic enzymes including activity, solubility and stability. In addition, she investigates enzyme kinetics under these conditions. She has shown that water and ion binding to halophilic proteins is significant in their function. Also, Ebel was involved in elucidating crystal structures of several halophilic enzymes (Representative references: (Ebel et al., 1999, 2002; Madern et al., 2000).

#### Sabrina Fröls

Complex microbial communities, i.e., biofilms formed by archaea and bacteria are recognized to be the predominant microbial mode of life in nature and found in a remarkable spectrum of habitats. Halophilic biofilm forming archaea were identified from sediments around an underwater fresh spring in the Dead Sea. Selected haloarchaeal strains of five different genera were tested in regard to surface adhesion. Fröls et al. (2012) showed, also by microscopic analyses, that this ability is widely distributed in halophilic archaea. The observed biofilms varied in architecture. Biofilm composition analyses revealed extracellular polymeric substances (extracellular DNA and glycoconjugates). By transmission electron microscopy studies of attached *Halobacterium salinarum* they observed multiple pili structures which might be involved in the haloarchaeal biofilm formation (Representative references: Fröls et al., 2012; Ionescu et al., 2012; Fröls, 2013).

#### Nina Gunde-Cimerman

Gunde-Cimerman discovered halophilic and halotolerant fungi, particularly melanized black yeasts and related fungi as inhabitants of solar salterns and other hypersaline environments around the world. Besides describing their biodiversity, including new species, she focused on molecular mechansims of adaptation to hypersalinity. Along with Ana Plemenitaš she studied physiological and molecular mechanisms of three model organisms: halotolerant *Aureobasidum pullulans*, extremely halotolerant *Hortaea werneckii* and the obligate halophile *Wallemia ichthyophaga*, at the level of cell wall, membranes, compatible solutes and expression of selected genes and lately also by whole genome sequencing (Representative references: Gunde-Cimerman et al., 2000; Plemenitaš et al., 2008; Zajc et al., 2013).

#### Inga Hänelt

The first response to an osmotic upshift and the immediate loss of water usually is the fast accumulation of K^+^ via channels, pumps and transporters. Hänelt’s research focuses on the functional role and molecular architecture of K^+^ translocating systems, in particular the bacterial Ktr system belonging to the superfamily of potassium transporters (Ktr/Trk/HKT family). Hänelt and others identified a unique flexible linker (loop) within the translocating subunit of Ktr/Trk systems that is crucial for the controlled uptake of potassium. The loop was shown to function as molecular gate that opens for the passage of K^+^ and is missing in classical K^+^ channels (Representative references: Hänelt et al., 2010a, 2010b, 2011).

#### Julie Maupin-Furlow

Prior to completion of any genome sequences of halophilic archaea, Maupin-Furlow and coworkers performed the groundbreaking experiments that demonstrated 20S proteasomes that catalyze proteolysis are expressed in the haloarchaeon *Haloferax volcanii*. They were purified, biochemically characterized and sequenced. Maupin-Furlow and co-workers showed that 20S proteasomes were not restricted to species of *Thermoplasma* and that they could be disassembled and reassembled *in vitro* based on salt concentration. This was the first observation of this kind for archaea. Maupin-Furlow also contributed to the characterization of the ubiquitin-fold proteins of the model archaeon *Haloferax volcanii.* Distribution of proteins of the ubiquitin-fold superfamily suggests sampylation is universal to Archaea. This study provided a fundamental insight into the diverse cellular functions of the ubiquitin-fold superfamily and the capacity for an archaeal ubiquitin-activating enzyme E1 homolog to have broad substrate specificity (Representative references: Wilson et al., 1999; Humbard et al., 2010; Miranda et al., 2011).

#### Noha Mesbah

Mesbah’s research is focused on poly extremophiles: halophiles, alkaliphiles, and thermophiles – on their isolation, identification, and characterization of adaptive strategies that allow them to survive and grow in the presence of multiple environmental extremes. She described a novel order, *Natranaerobiales*, with three obligately anaerobic, halophilic alkalithermophilic bacteria: *Natranaerobius thermophilus, N. Trueperi*, and *Natronovirga wadinatrunensis*. *N. thermophilus* has been used as the model microorganisms. It acidifies its cytoplasm, maintaining a constant transmembrane pH gradient of approximately 1 unit, acid inside. The genome of *N. thermophilus* was sequenced. Analysis showed an unusually large number of cation-proton antiporters, which aid to withstand multiple environmental extremes (Representative references: (Mesbah et al., 2007; Mesbah and Wiegel, 2011; Zhao et al., 2011).

#### Melanie Mormile

Mormile’s research on saline systems has ranged from studying the microbial ecology in hypersaline lakes to the development of methods for the retrieval of viable bacteria from ancient salt crystals. She isolated *Halomonas campisalis* from the region around Soap Lake in Washington State, with the aim to treat saline, alkaline waste. Further isolates from Soap Lake included a new genus, *Nitrincola lacisaponensis*, and a bacterium, *Halanaerobium hydrogeniformans*, capable of significant hydrogen production from alkali-treated cellulosic biomass. Along with K. Benison and F. Oboh-Ikuenobe she studied the microbial communities in the acidic saline lakes of western Australia. She also retrieved a viable *Halobacterium salinarum* cell from a 97 kyr halite crystal fluid inclusion. This work has led others to further refine the techniques used to retrieve microorganisms from evaporative crystals much older that 97 kyr (Representative references: Mormile et al., 1999, 2003, 2009).

#### Francisca Oboh-Ikuenobe

Oboh-Ikuenobe studied sediments and waters of different acid, neutral, and alkaline hypersaline ephemeral lakes in Western Australia, utilzaing sedimentological, geochemical, mineralogical, microbiological and palynological techniques. The aim was to identify microorganisms trapped in evaporites and/or sediments, to gather paleoenvironmental and paleoclimatic information and make analogies with Mars. Her group identified novel Bacteria/Archaea and algae and found microfossils in halite, gypsum, and hematite precipitates. Oboh-Ikuenobe’s focus was mainly on palynology. She identified *Dunaliella* as a proxy of arid climatic conditions and high concentration of salt in the region’s geologic record (Representative references: Benison et al., 2007; Bowen et al., 2008; Sanchez Botero et al., 2013).

#### Felicitas Pfeifer

Pfeifer worked on *Halobacterium salinarum* (*halobium*) already in the late 1970s and characterized the genetic variability of the large plasmids that incurred insertions and deletions due to insertion elements. She isolated many of these ISH elements from purple membrane (*bop*) mutants, recognized a transposition burst of the ISH27 insertion element family, and presented data on an AT-rich island in the genome of *Halobacterium salinarum* PHH1 harboring many insertion elements. Pfeifer was involved in the construction of vector plasmids to manipulate haloarchaea, and a vector carrying the origin of replication of plasmid pHH1 was used to demonstrate by that a fragment as large as 11 kbp is required for gas vesicle formation. These proteinaceous structures enable the cells to increase buoyancy and float to the surface of the brine. Pfeifer and coworkers demonstrated that a total of 14 *gvp* genes arranged in two clusters are required for gas vesicle formation in *Halobacterium salinarum* and *Haloferax mediterranei*. Gas vesicle formation is influenced by environmental factors such as salt concentration, oxygen availability, and light and thus serves as a model system to study signal transduction pathways in haloarchaea (Representative references: Pfeifer and Betlach, 1985, Pfeifer and Blaseio, 1990; Pfeifer, 2012).

#### Ana Plemenitaš

Plemenitaš’ lab together with Gunde-Cimerman lab focused on molecular mechansims of adaptation to hypersalinity, particularly in the extremotolerant model organism black yeast *Hortaea werneckii.* The adaptations were studied on the level of membrane composition and fluidity, ion pumps, differential gene expression at high and low salinity and selection of target genes (for example HAL) for transformation of yeast and plants to increase their halotolerance. The main focus of her lab is the study of high osmolarity gylcerol signaling pathway (HOG) – identification of the components, their interconnectivity and differences in selected halotolerant and halophilic fungal model organisms (Representative references: Lenassi et al., 2007, 2013; Vaupotiè and Plemenitaš, 2007).

#### Mecky Pohlschroder

A significant portion of the prokaryotic proteome is composed of secreted proteins, which play crucial roles in a variety of vital cellular processes, including nutrient uptake, cell wall biosynthesis, conjugation and motility. Pohlschroder focuses on characterizing the protein translocation pathways that transport these proteins to the cell surface. Computational searches performed by Pohlschroder suggested that several aspects of the universally conserved Sec translocation pathway are unique to archaeal species. Although most prokaryotic proteins are secreted in an unfolded conformation via the Sec pathway, nearly half of haloarchaeal secreted proteins are transported in a folded conformation via the twin arginine transport (Tat) pathway, as an adaptation to the high salt environments. Pohlschroder’s lab has developed several computational programs that can accurately predict the transport pathways used by specific substrates as well as the subcellular localization of secreted archaeal proteins (singalfind.org). Most recently, her lab identified a previously unknown cell surface anchoring mechanism. These investigations will also elucidate molecular mechanisms that support crucial biological processes in prokaryotes, as illustrated by the variety of phenotypic defects identified by the Pohlschroder lab in an *Haloferax volcanii* archaeosortase deletion mutant, which include deficiencies in cell wall biosynthesis, as well as surface adhesion, mating and motility (Representative references: Pohlschroder et al., 1997; Rose et al., 2002; Abdul Halim et al., 2013).

#### Emilia Quesada

Quesada was one of the pioneers, together with Antonio Ventosa, in the study of halophilic bacteria inhabiting hypersaline waters. They developed different techniques for their cultivation and identification. She also made the first studies of saline soils and described its microbiota (bacteria and halophilic archaea). She discovered many species of halophilic bacteria and became a member of the International subcommittee on the *Halomonadaceae* family. Her lab described for the first time systems of quorum sensing in halophilic bacteria. They also found and developed a number of exopolysaccharides produced by halophilic bacteria of biotechnological interest (Representative references: Mata et al., 2008; Amjres et al., 2011; Luque et al., 2012).

#### Elina Roine

The general objective of Roine’s research is to characterize viruses from scarcely studied niches, to increase information of the functions of genes residing in prokaryotic viral genomes and virus related functions in their respective hosts. Her main impact in haloarchaeal research has been detailed characterization of new viruses, such as the description of the first ssDNA archaeal virus and a new family of pleomorphic viruses. She showed that the genomes of haloarchaeal pleomorphic viruses differ and that the structure of their major N-glycan modifiying spike protein is involved in the host interaction. On the basis of these studies Roine and co-workers suggested a wider evolutionary connections between the pleomorphic viruses and a group of bacteriophages. She also studied the icosahedral virus SH1 and, together with Angela Corcelli, the lipids of haloarchaeal viruses (Representative references: Pietilä et al., 2009; Kandiba et al., 2012; Senèilo et al., 2012).

#### Shereen Sabet

Sabet was the first to describe the viral assemblage in the hypersaline Mono Lake with a metagenomic approach. Although her goal was to clone an entire viral genome from an environmental sample, she was nevertheless able to clone several fragments. She was the first to isolate viruses from Exportadora de Sal in Baja California, Mexico. The genome of one of those viruses, GNf2, has been sequenced. She also undertook a comparative metabolic analysis on the archaeal and bacterial isolates from ESSA, similar to cultures that had been isolated from the same site 24 years prior by Barbara Javor. She showed adaptive micro-environmental signatures of substrate usage within the halophile population. Shereen’s most recent contribution is isolation of viral plaques from the Great Salt Lake and the Cargill solar salterns in San Francisco Bay (Representative references: Sabet et al., 2006, 2009; Sabet, 2012).

#### Helena Santos

Santos’s work is focused on the understanding how marine hyperthermophilic microorganisms adapt to life at temperatures around 100^°^C, which are lethal to so many other cells. Microorganisms isolated from marine habitats are slightly halophilic and, like many halophiles, accumulate compatible solutes for osmoregulation. Santos and others found that hyperthermophiles accumulate exquisite organic solutes, such as mannosylglycerate and di-*myo*-inositol-phosphate, which are used for osmoprotection and also for thermoprotection. They identified several novel solutes, characterized their biosynthetic pathways, determined the 3D-structures of biosynthetic enzymes, studied their physiological role using suitable mutants, probed the molecular basis for protein stabilization, and searched for novel applications (five international patents). Amongst Santos’ recent achievements is the establishment of the role of ionic solutes in thermoprotection using mutants and evolution of the biosynthesis of di-*myo*-inositol phosphate. Her group also established the role of mannosylglycerate in the stabilization of proteins, via restriction of the slow motions of specific structural elements (Representative references: Rodrigues et al., 2007; Faria et al., 2008; Pais et al., 2012).

#### Amy Schmid

Schmid’s overarching research goal involves understanding the molecular mechanisms underlying the transcriptional response of halophilic archaea to environmental changes and how this results in physiological adaptation. Central to this process are gene regulatory networks (GRNs) composed of groups of interacting transcription factors (TFs) and their target gene promoters. Upon sensing a change in the environment, signal transduction cascades propagate the information to GRNs, where TFs proteins induce genes encoding proteins that restore the cell to a stable state and prepare for future stress. In halophilic archaea, and archaea generally, the molecular function of TFs is unclear relative to the other domains of life. Schmid uses *Halobacterium salinarum* as a model system for understanding how GRNs function dynamically to survive during strong daily fluctuations in conditions within arid hypersaline environments, which can result in extreme oxidative damage to macromolecules. In response, TFs induce genes encoding enzymes to neutralize oxidants, repair damaged molecules, and restore redox balance in the cell. In more recent work, the Schmid group has focused on validating subsets of the GRN composed of 2–3 interacting TFs using integrated genome-scale TF-DNA binding and gene expression analyses (Representative references: Schmid et al., 2011; Sharma et al., 2012; Todor et al., 2013).

#### Helga Stan-Lotter

Stan-Lotter initially focused on the ATPase enzymes of halophiles for energy transduction. Archaeal enzymes were found to have a similar structure as those from non-Archaea, but there were enough differences to put them into a separate class. In the Austrian alps, she isolated viable extremely halophilic Archaea from rock salt deposits of Triassic and Permian age. Taxonomic characterization showed that some properties of the isolates were similar to those of known haloarchaea; however, numerous differences suggested that the strains were novel species. These microorganisms may have survived enclosed in the fluid inclusions of rock salt since the evaporation of ancient brines. Repeated re-isolation of the same strains from the same sites suggested authenticity of the isolates. In preparation for the search for extraterrestrial life in halite on planets like Mars, Stan-Lotter provided a halophile strain isolated from Permian salt, *Halococcus dombrowskii*, for exposure of extremophiles on the outside of the International Space Station. This strain was chosen due to its resistance to desiccation and irradiation (Representative references: Stan-Lotter et al., 1999; McGenity et al., 2000; Fendrihan et al., 2009).

#### Nicole Wagner

Wagner initially studied optimization of retinal containing proteins for application in devices and played an integral role in the proof-of-concept studies that helped to found in 2009 LambdaVision Incorporated. Via site-directed mutagenesis, site-specific saturation mutagenesis, and directed evolution she was able to genetically engineer BR, for application in a number of device architectures, Most recently, a protein-based retinal implant, which is targeted at restoring vision to patients with age-related macular degeneration and retinitis pigmentosa. BR, located in the outer membrane of *Halobacterium salinarum*, functions as a light-transducing proton pump. The native protein has a photocycle, which is made up of a series of transient photochemical and conformational intermediates. It is this unique photocycle that makes BR one of the most studied proteins for use in bioelectronics and biomimetic devices (Representative references: Greco et al., 2012; Wagner et al., 2013a, b).

#### Tatjana Zhilina

Zhilina, with coworkers was involved in the isolation, identification and description of new species of halophilic bacteria from sediments of soda lake Magadi (Kenya), amongst them a novel genus and species, *Natranaerobaculum magadiense* gen. nov., sp. nov. and from sediments of the soda-depositing soda lake Tanatar III (Altay, Russia) of the order *Halanaerobiales*, which represented a new branch within the family Halobacteroidaceae. They described a novel species in a new genus with the name *Fuchsiella alkaliacetigena* gen. nov., sp. nov. They also obtained new isolates from denitrifying enrichments with various electron donors using sediment samples from hypersaline soda lakes. They were identified as members of the *Gammaproteobacteria* closely associated with the *Alkalispirillum-Alkalilimnicola* group and demonstrated much higher metabolic diversity of haloalkaliphilic *Gammaproteobacteria* than was originally anticipated (Representative references: Sorokin et al., 2006; Zhilina et al., 2012; Zavarzina et al., 2013).

### ANALYSIS OF HALOPHILE CONFERENCE PARTICIPATION 1978–2013

**FIGURE 1:**
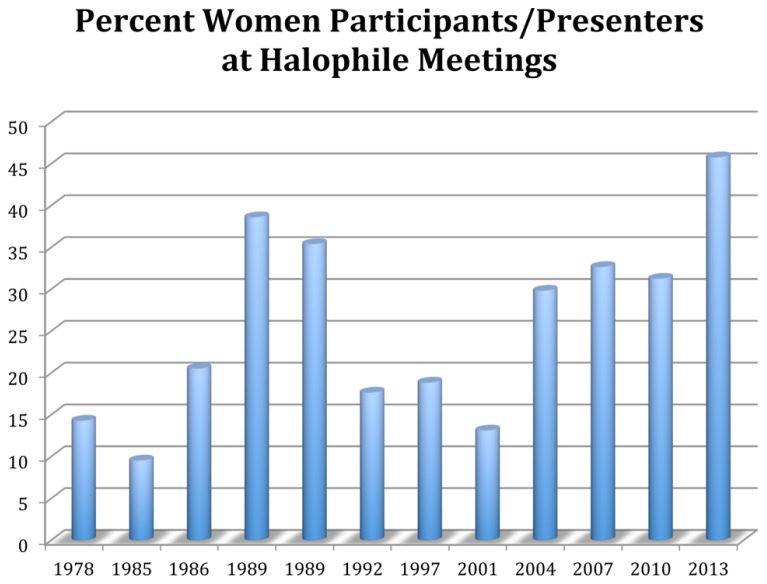
**Participation of women as speakers in international halophile conferences from 1978 until 2013**.

International symposia for halophile microbiologists have been held with some regularity since 1978 (Oren, 2011). Conference programs for 12 international Halophiles meetings (**Table 1**) were analyzed. For all meetings, we noted female presenters as well as female membership of the organizing committee. We examined the rosters of *all participants* for conferences held 1978 through 1997 since the numbers of attendees was rather low and the programs were arranged in a way that did not distinguish the type or presentation. However, for the meetings during the years 2001–2013, we only looked at the oral presentations. This count revealed a steadily growing number of women over time, with the exception of the two conferences in 1989, which seemed heavy in female participation when compared to the years before and after this date (**Table 1** and **Figure 1**. The high numbers of female speakers for 2013, 46%, was an unanticipated result and deserves some analysis as this is out of synch with reports from other fields (Eisen, 2012; Isbell et al., 2012; Schroeder et al., 2013).

The halophile conferences that had at least one woman as a member of the organizing committee (1985, September, 1989, 2004, 2007, and 2013) show higher numbers of female presenters with the exception of the earlier date, 1985, where the women gave only 9% of the presentations. If we leave out the data from 1985, these other conferences show an average of 36% women giving talks. The number drops to 30% if the data set for 1985 is included. In contrast, the conferences that did not have female convener membership had an average of 20% female presenters.

## DISCUSSION

The underrepresentation of women in science has been well documented in the literature. Several studies show societal factors that are at play (Shen, 2013), and gender biases in various fields have been identified (Conley and Stadmark, 2012; Eisen, 2012; Schroeder et al., 2013). Indeed, women have gained ground in science, but there are many gaps still to close (Shen, 2013).

Halophile science is rather interdisciplinary as participants may work in the lab and/or field and be a part of microbial ecology, biochemistry, geology, or even planetary science communities. Therefore, this population of scientists should represent a broad sampling with which to ask the question of gender bias. Given the male-dominated sub-fields, we predicted that women would be underrepresented in halophile science.

### HALOPHILE SCIENTISTS: AN EXAMPLE OF PROGRESS

Historically, many women scientists have been engaged in halophilic microbiology. A few notable examples, including Yonath’s Nobel Prize and Colwell as the President of the U.S. National Science Foundation, indicate that there were indeed amazing female contributors as this field was developing. In recent years, the numbers of women participating in the international congresses has generally increased.

Among the successes we encountered, the authors of this study also uncovered stories of discrimination as we polled women currently working in halophile research. Several very accomplished female scientists noted that their discoveries had been overshadowed by those of men in the field. Certainly no field of science is without bias and discrimination as it is an endeavor of humans. Halophile science is no exception, but by and large, here we report some very positive outcomes.

The most significant moment in this study was the end of our presentation for opening event at the 2013 Halophiles Meeting in CT, USA, where we announced that 46% of the speakers for the conference would be women. This was unexpected for us as we calculated the results, and it was unanticipated for our audience. It seems the population of salty scientists are doing better than other fields if representation of women in lectures is any measure (Conley and Stadmark, 2012; Eisen, 2012; Schroeder et al., 2013). Why would this particular field of study lead to less gender bias than other fields have observed?

**FIGURE 2:**
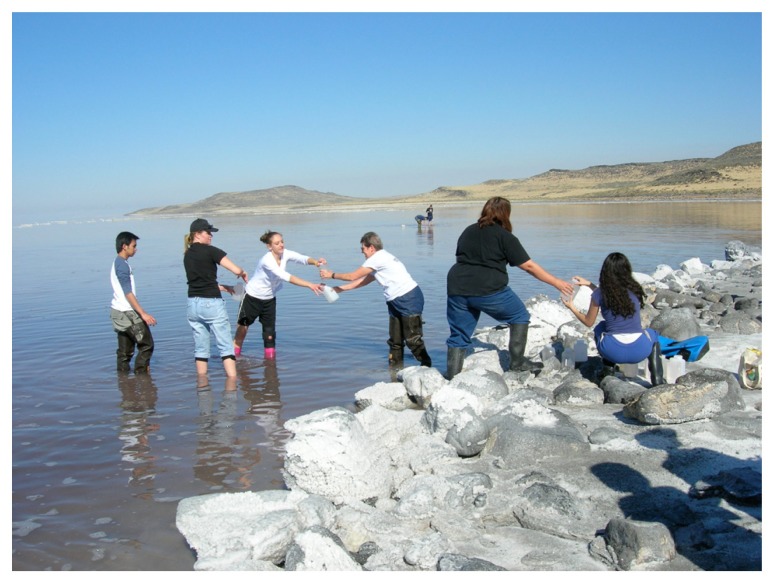
**Cooperative Fieldwork**. Carol Litchfield, center, leads a sampling brigade at Great Salt Lake, Utah, USA.

Discussions among colleagues revealed an important point: halophile microbiologists who attend the international congresses understand that they are in a field where mentorship is valued. This has historically been a small group of scientists, committed to holding conferences despite no professional organization and no secure funding. Strong friendships were formed, and many of the participants began collaborations that spanned years, working side by side in the laboratory or the field (**Figure 2**). Also, in more than a decade, organizing committees have made the invitation of young scientists and in particular, women, a mission of the meetings. This philosophy of inclusion and even actively providing travel funds for underrepresented speakers has ensured an almost equal gender representation.

Perhaps placement of women on the organizing committees for the international halophile congresses has allowed for more inclusion. When we analyzed the conference data we saw a 10–16% increase in female speakers when at least one woman was included among the conveners. This echoes the study by Casadevall and Handelsman (2014) that showed as much when looking at a much broader sample of American Society of Microbiology symposia. Indeed, the gender balance and role of the organizing committee for any conference cannot be overlooked and may provide a significant impact on representation of women. Halophile scientists have employed this strategy often, and we suggest that this becomes a continued practice. We are a community that encourages the participation of women by inclusion among the conveners, resulting on average a higher number of female speakers on average than in other fields.

We propose that the model of inclusion and mentorship experienced in our field should continue through concerted efforts, and it should be applied more broadly to all fields of science. To extrapolate from a famous quote by Marie Curie: after all, science is without *genders*, and it is only through lack of the historical sense that *gender* qualities have been attributed to it.

## Conflict of Interest Statement

The authors declare that the research was conducted in the absence of any commercial or financial relationships that could be construed as a potential conflict of interest.
